# A new highly digestible prescription diet containing *Bacillus velezensis* DSM 15544, fructo-oligosaccharides, plasma immunoglobulin, yeast and sepiolite for the management of acute diarrhea in dogs—a randomized double-blinded, controlled trial

**DOI:** 10.3389/fvets.2025.1665730

**Published:** 2025-12-15

**Authors:** I. Jeusette, E. Apper, V. Fragua, N. Sanchez, C. Torre, L. Badiella, J. Puig, E. Ballester, A. Jurado, L. Feo Bernabe

**Affiliations:** 1 R&D Department, Affinity Petcare, Barcelona, Spain; 2 ImProVet, Barcelona, Spain; 3 Mathematics Department, Autonomous University of Barcelona, Barcelona, Spain; 4 Ars Veterinary Hospital, Barcelona, Spain; 5 AniCura Ars Veterinaria Hospital Veterinari, Barcelona, Spain

**Keywords:** dog, acute diarrhea, probiotics, prebiotics, diet, animal plasma proteins

## Abstract

**Objective:**

To determine if a newly formulated diet with safe and highly digestible ingredients, *Bacillus velezensis* DSM 15544, fructo-oligosaccharides, animal plasma protein, dried whole yeast and sepiolite contributes toward the management of canine acute diarrhea.

**Hypothesis:**

The new diet (Diet B) will reduce the time to achieve normal fecal consistency compared to a highly digestible control (Diet A).

**Methods:**

Multicenter randomized, double-blinded, parallel-designed study with adult dogs with mild to moderate acute signs (less than 7 days) of uncomplicated diarrhea as inclusion criteria. Exclusion criteria were history of gastrointestinal signs, lack of correct vaccination and deworming, abnormal ultrasound examination, and previous administration of antibiotics, omeprazole or nutritional supplement. Response variables were compared using the appropriate bivariate test, time to recovery was analyzed using survival analysis techniques.

**Results:**

One hundred eleven dogs finished the study. Dogs fed Diet B (*n* = 56) had a quicker recovery time of fecal consistency compared to A (3.6 ± 0.9 vs. 5.9 ± 0.9 days). Fecal frequency and odor were also quickly restored compared to diet A. Survival analysis confirmed a 1.6 times greater chance of recovery with Diet B compared to diet A. Feeding Diet B also improved dysbiosis index at recheck compared to diet A (−1 ± 3 vs. 0.65 ± 3).

**Conclusion:**

Diet B resulted in faster clinical resolution, improved fecal consistency, frequency, odor and reduced incidence of dysbiosis compared to Diet A, making it a superior dietary approach in the management of mild acute diarrhea in dogs.

## 1 Introduction

Acute diarrhea is one of the most common reasons for veterinary consultations in dogs in Western countries. Reports indicate that approximately 10 to 20% of dogs visiting primary veterinary practices present with gastrointestinal disorders (1–4). Although acute diarrhea tends to be self-limiting in most cases, and with a mild impact on dog well-being, owners commonly seek veterinary consultation (5). There are several causes of acute diarrhea, including gastrointestinal and extra intestinal conditions. Some of the most common causes are dietary indiscretion, sudden dietary changes, stress, medications such as non-steroidal anti-inflammatory drugs and gastrointestinal infections (e.g., *Escherichia coli*) (6, 7). Etiological treatments are rarely established because the exact cause is often difficult to identify (8). Currently, antimicrobials are typically not recommended (9). The replacement of antimicrobials with alternative approaches aimed at restoring gut microbiota, such as biotics and/or dietary modifications, is now a therapy to consider for dogs suffering from acute gastrointestinal disorders (7, 10–13). Intestinal dysbiosis is characterized by an imbalance in bacterial composition, metabolic activity, and/or distribution within the gut that is associated with disease (14). It is commonly assessed using the Dysbiosis Index (DI), a commercially available scientifically validated PCR-based assay that quantifies core bacteria and accurately predicts global microbiome shifts in individual patients. A DI < 0 indicates no change in overall microbiota diversity, a DI between 0 and 2 indicates a mild to moderate dysbiosis and a DI > 2 signifies significant intestinal dysbiosis (15–17). This increase in DI may result from the use of antibiotics and may be observed in acute (generally mild and transient) and chronic diarrhea in dogs (15–18).

The development of a complete and balanced diet designed to accelerate the recovery of dogs suffering from acute diarrhea can be a valuable tool for veterinarians, limiting discomfort for both the dog and the owner. To this end, we developed a diet based on safe, approved, and highly digestible ingredients and nutraceuticals. Specifically, the nutraceutical components included one probiotic strain, fructo-oligosaccharides, spray-dried porcine plasma (SDPP), dried whole brewer yeast and sepiolite.

Probiotics are defined as live microorganisms that, when administered in adequate amounts, confer a health benefit on the host (19). *Bacillus velezensis* DSM 15544 (formerly known as *B. subtilis* DSM 15544) is a live probiotic approved by the European Food Safety Authority (EFSA) for dogs and regarded as suitable for the qualified presumption of safety (QPS) (Table 1) (20). This indicates that the strain is conclusively established, non-toxigenic, and shows no resistance to antibiotics of human and veterinary importance. It has been reported to have several beneficial effects in canine studies, including improved fecal consistency, enhanced gut microbiota diversity, increased fecal IgA levels, and reduced serum ammonia and C-reactive protein levels (20, 21). The stability of *Bacillus velezensis* DSM 15544 in extruded kibbles for a minimum of 18 months has been recently demonstrated (22). SDPP is a well-known source of protein and functional ingredients in the petfood industry (23). It has been demonstrated that orally administered immunoglobulins (IgG) from SDPP retains its activity throughout the canine digestive tract. This implies that it can confer passive immunity at the intestinal level, thereby enhancing the dog’s natural defenses (24). Furthermore, *in vitro* research has indicated that IgG from SDPP can bolster gut immunity. A study using canine epithelial cells revealed decreased adherence and invasion of enteropathogens when exposed to SDPP-derived IgG, suggesting a potential protective role in the canine gut (25). In addition, numerous *in vivo* studies have reported a positive effect of SDPP on the intestinal morphology and immune systems of various animals, including fish (26), pigs (27), mice (28), and poultry (29). A recent study evaluated the inclusion of *Bacillus velezensis* DSM 15544, with and without SDPP, in healthy dogs showed that compared to placebo, dogs fed this mix had higher fecal concentrations of short chain fatty acids, higher relative abundance of beneficial taxa such as *Faecalibacterium prausnitzii*, lower relative abundance of potentially harmful bacteria like *Prevotella copri*, higher fecal IgA contents and higher blood leukocyte counts, particularly lymphocytes and neutrophils, although the addition of porcine plasma did not seem to confer additional benefits in this study (30).

**Table 1:** Description of the probiotic strain (*Bacillus velezensis* DSM 15544, formerly known as *B. subtilis* DSM 15544) tested in the study.

Strain	Safety	Authorized in the EU market	Dose for use as per the EU authorization	Stability in extrusion
*B. velezensis* DSM 15544 (formerly *B. subtilis* DSM 15544)	QPS (EU)	For dogs	1×10^9CFU/kg petfood	Demonstrated for a shelf-life of 18 months in kibbles (22)

Prebiotics (e.g., fructo-oligosaccharides, FOS) are indigestible fibrous compounds that stimulate the growth of beneficial intestinal bacteria, such as *Bifidobacteria* and *Lactobacillaceae* family, reduce the concentration of putrefaction compounds in feces, and improve gut health and the smell of the feces (31, 32). FOS (0.4% in this study) have also been tested in several studies in dogs (dose from 0.2 to 1%), showing safe prebiotic effects and a good digestive tolerance up to 1% (33, 34).

Yeast ingredients are common in dog food. There are different forms of yeast products, ranging from viable yeast cells to purified components of the cell wall. In this study, dried whole brewer yeast (1%) was used. Whereas dried yeast is used for its nutritional value (amino acid, vitamins and minerals), it is still a source of beta-glucans and mano-oligosaccharides (MOS) that can improve gut health and enhance immune system (35).

FOS, SDPP and yeast are ingredients recognized as raw materials as per the EU regulation 767/2009 (36) and are registered under the EU feed catalog 68/2013 (37).

Sepiolite is an adsorbent additive that helps maintain good fecal scores, improves fecal characteristics, and reduces odor (38, 39). The inclusion of sepiolite is also considered as safe by EFSA in all animal species including dogs (maximum recommended level of 20,000 mg/kg at 12% moisture) (40).

The main goal of the study was to determine if a new Diet (Diet B) contributes toward the management of canine acute diarrhea. The hypothesis was that this new diet could decrease the time to normalization of fecal consistency (defined as one entire day with no fecal score ≥ 2) when compared to a non-commercial diet (Diet A), specifically formulated to be highly digestible. Diet B included highly digestible ingredients, probiotic (single strain of *Bacillus velezensis*), prebiotic (FOS), SDPP protein, dried whole yeast and sepiolite, while Diet A served as a highly digestible control, differing from the Diet B on its main ingredients and without the nutraceuticals. The study assessed both clinical improvement and changes in fecal DI to determine whether the supplemented diet offered significant therapeutic advantages.

## 2 Materials and methods

### 2.1 Study design

The study was a multicentric, randomized, double-blinded, controlled, parallel-designed study conducted in five veterinary practices in the Barcelona area with client-owned dogs from November 2023 to February 2024. Affinity Petcare ethical committee approved the protocol (RS000371).

**Figure 1:**
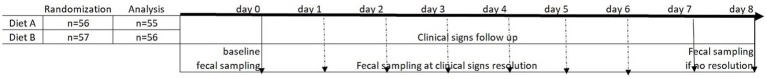
Study design.

Owners were orally informed by the veterinarian about the essential study information (objective of the study, procedures to be implemented, clinical signs follow up) and an informed consent form in the local language was signed by all owners before inclusion of their dog in the study to confirm the participation of his/her animal. By signing the form, the owners accepted to answer a survey at enrolment (baseline) and recheck, to report the clinical signs (primarily documenting all defecation events), to attend visits at the beginning and end of treatment, and provide a fecal sample from their dog at enrolment and after clinical signs resolution (or after 7 to 8 days if clinical signs did not resolve before) for dysbiosis index evaluation (Figure 1, study design). If clinical signs or dysbiosis persisted after 7–8 days, pet owners were offered the option to switch their dog’s diet to another diet (Diet GE, equivalent to the Test diet), when possible and continue the follow-up as described above.

Clients were also informed that in exchange for providing a proper follow-up, they would receive the food and have the clinical exams free.

The dogs that met eligibility criteria (inclusion and exclusion criteria, see below) were selected by the veterinarians and then referred to the coordinating veterinarian the same day to assign the dietary treatment. The dogs were randomly allocated to one of the diets (Control, Diet A, and Tested Diet, Diet B) in a 1:1 ratio using a randomization list generated by statistical software prior to participant enrollment and were fed for a period of 8 days. All individuals involved with the animals (veterinarians, coordinating veterinarian, owners) or with the data (data reporter, statistician) were blinded to the treatments but no formal allocation concealment method (e.g., sealed opaque envelopes) was used in this study. On the first visit, the coordinating veterinarian determined the recommended food intake based on the dog’s body weight and activity level (feeding guideline provided by one of the coauthors not involved in dogs’ recruitment) (38). On the second day after the study began, a follow-up call was made to ensure the dogs’ conditions were not worsening and that everything was understood, agreed upon, and applied. The coordinating veterinarian of the study was always reachable by phone or by chat if needed.

#### 2.1.1 Inclusion criteria

Dogs receiving regular anti-parasitic treatments and vaccinations were eligible for this study. Eligibility criteria evaluated by the attending clinician included first episode of mild to moderate acute signs (<7 days) of diarrhea with or without concurrent vomiting, a body weight ranging from 4 to 45 kg, being over 8 months of age, and having no clinically relevant comorbidities that could be expected to cause secondary diarrhea (such as endocrinopathies, organ dysfunction, immune-mediated diseases, or suspected pancreatitis indicated by severe abdominal pain). Diarrhea was defined as a fecal consistency score ≥2 on a 7-point adapted Bristol stool chart (41, 42), with score from −2 to 4, where a negative score indicates hard feces, 0 and 1 represent ideal stools and 2 to 4 indicates mild to severe diarrhea (Table 2).

**Table 2:** Scale used to estimate fecal consistency (adapted from Bristol stool chart) (42).

Bristol stool chart	Trial simplified score	Fecal consistency		Description
1	−2	Unhealthy (Constipation)	Hard and dry	Hard and dry, in the form of separate lumps. The dog often has difficulty expelling stools. No residue on the ground when collected.
2	−1		Firm and slightly hard	Firm and slightly hard, cylindrical in shape. Visible cracks on the surface. Dry surface, but slightly moist inside. Little or no residue on the ground when collected.
3	0	Healthy	Firm and well-formed	Firm, with a slightly damp surface. Cylindrical shape, with some visible cracks. Leave a slight residue on the ground.
4	1		Firm and moist	Firm and moist, but they lose their shape when collected. Cylindrical shape, no visible cracks. Leave residue on the ground.
5	2	Unhealthy (Diarrhea)	Pasty with some shape, soft	Pasty and wet. Still with some cylindrical shape but piled up. Do not maintain their shape when collected. Leave residue on the ground.
6	3		Pasty, shapeless	Pasty texture, non-cylindrical form. Feces is piled up and partially liquid. Leave residue when collected and difficult to pick up.
7	4		Liquid	Liquid texture, without any form. No solid pieces. Very difficult to collect.

#### 2.1.2 Exclusion criteria

Dogs that presented signs of liver, kidney, and/or pancreatic diseases, or foreign bodies during an abdominal ultrasound exam were excluded. Similarly, dogs that had received antibiotics, omeprazole, or nutritional supplements within the 4 weeks preceding the study’s start date were also excluded. Dogs had to be withdrawn from the study in case of owner non-compliance with diet administration, or the presence of extra gastrointestinal conditions or other severe clinico-pathological alterations (43, 44). Body condition score (BCS) on a 5-point scale was recorded but was not a criterion for inclusion or exclusion. The BCS was not considered as an inclusion/exclusion criterion because we were targeting acute diarrhea, so we were not expecting impact from and on BCS at a very short term and dogs with any BCS could suffer from acute diarrhea. Eligibility, along with complementary exams, was at the discretion of the attending clinician at the time of presentation.

### 2.2 Diets

The two diets were specifically formulated for the study with different highly digestible ingredients and were not commercially available at the time of the study (Table 3). The tested diet (Diet B) included among others the following functional ingredients: prebiotic (0.4% FOS), probiotic (minimum 1.0 ×10^9^ CFU/ Kg *Bacillus velezensis* DSM 15544), 1% plasma immunoproteins, 1% yeast (with approximately 16% beta-glucans and 6% MOS) and 0.5% sepiolite (Table 3). In term of ingredients, Diet B contained more rice, more corn, more vegetal protein, fish oil, coconut oil and cellulose compared to diet A. Diets were dried to a constant weight at 103° to determine dry matter (DM, ISO 1442, 1997). Crude ash values were determined by combustion at 550° (ISO 935, 1998). Crude protein was calculated from Kjedahl nitrogen (6.25xN, ISO 5983-1, 2005). Crude fiber was analyzed by acid- alkali digestion (ISO 5498, 1981) and crude fat was analyzed using acid-hydrolysis followed by Soxhlet extraction (ISO 1443, 1973). Nitrogen free extract was calculated subtracting crude ash, crude protein, crude fat, and crude fiber on DM basis from 100. Metabolizable energy (ME) was calculated using predictive equation following European Standard EN 16967 recommendations (38). All nutrients were expressed as g/1000 kcal calculated ME. *As per* analysis, Diet B contained more protein, less fat and crude fiber compared to Diet A (Table 3). Apparent digestibility of diets was previously tested following protocol from the European Pet Food Industry (FEDIAF), and no significant difference was observed between diets for protein, fat, and dry matter digestibility (Table 3) (38). The study sites received the diets and stored them securely in a location according to the storage conditions specified on the labels. The two diets were labeled with specific packaging to ensure that both the veterinarian and the animal owner were blinded throughout the study duration. The two diets were packed in white plastic bag and labeled as highly digestible diet A or highly digestible diet B. In addition, a third diet was prepared and labeled as highly digestible GE for the cases that did not resolve in 8 days and that wanted to continue the follow up.

**Table 3:** Main nutrients, ingredients and apparent digestibility of Diet A (Control) and Diet B (Test).

	Diet A—Control	Diet B—Test
Main ingredients (% as fed)		
Rice	10	29
Corn	6.1	22.1
Corn gluten meal	2.5	17.4
Poultry meal	12.6	10.1
Poultry fat	7.3	3.5
Chicken	21.5	-
Whole wheat	18	-
Peas	3.4	-
Turkey meal	3.3	-
Sugar beet pulp	2.9	-
Coconut oil	-	3.9
Hydrolyzed soy protein	-	1.8
Yeast	-	1
Fish oil	-	0.5
Sepiolite	-	0.5
Spray dry plasma protein (SDPP)	-	1
Fructo-oligosaccharides	-	0.4
Cellulose	-	0.3
Probiotics - *Bacillus velezensis* (CFU/kg)	-	Min. 1×10^9^
Main nutrients (g/1000Kcal ME*)		
Protein	65	71
Fat	42	34
Ash	17	18
Crude fiber	5.2	3.7
Total dietary fiber	21.9	12.8
ME (Kcal/kg DM)*	4,060	3,904
Apparent digestibility (%)		
Dry matter	83 ± 5	84 ± 2
Protein	84 ± 6	87 ± 2
Fat	96 ± 2	94 ± 3

*ME metabolizable energy calculated using NRC 2006 formula (Fediaf).

### 2.3 Animals

The veterinarian recorded the dog’s name, body weight, BCS, breed, sex, age and clinical signs in the corresponding case record form. Once included in the study, each dog was assigned a unique case identification code (dog ID).

### 2.4 Primary outcome

The current study’s primary outcome was the number of days to normalization of fecal consistency, defined as one entire day with fecal consistency score <2 for each dog.

Fecal consistency score was assessed using a slightly modified Bristol stool chart from −2 (hard feces) to 4 (severe diarrhea), with scores 0 and 1 being ideal (Table 2) (42).

### 2.5 Secondary outcomes

The following secondary outcomes were analyzed during the study

1. Duration of diarrhea analyzed through survival curve (proportion of dogs with fecal score ≥ 2 over time)
2. Percentage of dogs with clinical resolution within 8 days
3. Clinical secondary outcomes measured at each study days by the owners:

- Fecal consistency score (Table 2)
- Fecal frequency score, adapted from the scoring index for disease activity in canine inflammatory bowel disease (Table 4) (45)
- Fecal color using a binary scale (normal or not). Abnormal fecal color includes yellow, black, gray, white, and blood-stained patterns (Table 4). The presence of blood-stained patterns was analyzed separately for baseline evaluation.
- Fecal odor, using a binary scale (normal or not). Abnormal fecal odor includes putrid and sour milk scents (Table 4).

1. Body weight and BCS were measured by the veterinarian at baseline and at the end of the study.
2. Satisfaction surveys: At the end of the study, dog owners also had to complete a satisfaction survey (longitudinal scale from 0 to 10) regarding product palatability, facility to follow the dietary treatment, product satisfaction, product characteristics (odor, visual appearance, texture, color) and general satisfaction. They also needed to report whether they would recommend the diet and if they would repeat it in the event of a disease recurrence. At the end of the study, veterinarians from the different clinics were also required to complete a satisfaction survey (on a scale from 0 to 10) and indicate if they would recommend the same diet.
3. Fecal Dysbiosis index: A fecal sample was obtained for dysbiosis index evaluation via qPCR at T0 and after the resolution of clinical signs (16). If clinical signs were still present after 7 days, the fecal sample was collected 7 to 8 days after the initiation of diet. The DI was analyzed by a private laboratory (IDEXX, Barcelona) according to the method developed by AlShawaqfeh et al. Briefly, *Peptoacetobacter* (formerly *Clostridium*) *hiranonis*, *Faecalibacterium* spp., *Turicibacter* spp., *Streptococcus* spp., *E. coli*, *Blautia* spp., and *Fusobacterium* spp. were analyzed (log DNA/g) and used to calculate the index. According to the laboratory, a value < 0 indicates no dysbiosis; a value between 0 and 2 indicates a slight dysbiosis, while a value > 2 indicates severe dysbiosis (16).

**Table 4:** Scales used to estimate fecal frequency, odor and color scores.

	Fecal frequency		Fecal color	Fecal odor
Healthy	0	Normal (1–2 times/day)	Normal	Normal
Unhealthy	1	Slightly increased (3–4 times/day)	Not normal (i.e., yellow, black, white, gray and blood-stained pattern)	Not normal (i.e., putrid, sour milk scents)
	2	Moderately increased (5 times/day)		
	3	Severely increased (>5 times per day)		

The veterinarian assessed the fecal consistency score with the owner at baseline (day 0) and at the final sampling point, defined as the end of the clinical signs of diarrhea. In between the start and the end of the study, pet owners had to complete a daily leaflet until clinical signs resolved (or during 8 days in case of no resolution, day 0 being the baseline visit) with the parameters listed above: fecal consistency, defecation frequency, fecal color, fecal odor, and presence of vomiting.

The different primary and secondary outcomes are listed in Table 5.

**Table 5:** Primary and secondary outcomes measured during the trial.

Outcomes	Unit	Time of measurement	Results presentation
Primary outcome			
Number of days with clinical signs* (Fecal consistency ≥2)	Days	At the end	Table
Secondary outcomes			
Survival curve (% of dogs with fecal score <2 over time which means resolution of diarrhea)	%	Over 8 days	Figure (graph)
% of dogs with restauration of normal fecal score (<2) within 8 days	%	At the end	Table
Fecal consistency score	−2 to 4	Over 8 days	Figure (graph)
Fecal frequency score	0 to 3	Over 8 days	Figure (graph)
Fecal quantity	1 to 10	Over 8 days	Figure (graph)
Fecal odor	Normal/not normal	Over 8 days	Figure (graph)
Fecal color	Normal/not normal	Over 8 days	Figure (graph)
Body weight	Kg	At baseline and at the end	-
Body condition score	5-point scale	At baseline and at the end	-
Satisfaction surveys	Questioner	At the end	Table
Presence of dysbiosis, DI (and bacterias)	%, absolute number, log DNA/g	At baseline and at the end	Table

*Calculated from dogs that clinically resolved (fecal consistency <2) within 8 days.

### 2.6 Statistical power analysis

To detect differences between diets in the primary outcome of approximately 2 days, assuming a common standard deviation of 3 days, and using a Mann–Whitney U-test, a total sample size of 86 animals was calculated to be required. Considering potential dropouts, the required sample size was set to 100 animals. Sample size calculations were performed using the software Ene 2.0 (46). The standard deviation was estimated assuming a uniform discrete distribution [0, 10] for the primary variable. The sample size for a non-parametric test was determined from the sample size for a t-test applying the correction described by Hollander et al. (47).

### 2.7 Statistical analysis

Each parameter was first analyzed using appropriate bivariate tests to detect differences between groups. For quantitative variables, differences between groups were tested using an ANOVA or a Kruskal-Wallis test, considering as null hypothesis the equality between groups. The compliance of application criteria was assessed by means of Shapiro–Wilk’s normality test and Levene’s test for homogeneity of variances. Quantitative variables are summarized using mean, median, standard deviation, maximum, minimum, lower and upper 95% confidence interval limit. For qualitative variables, differences between groups were tested using a Chi-Square test, a Fisher’s exact test or LR Chi-Square test, considering as null hypothesis the equality between groups. The compliance of application criteria was assessed by means of the Cochran’s rule. Qualitative variables are summarized using absolute frequencies.

Then, in order to adjust the model and to refine the interpretation with the time, quantitative response variables (fecal consistency, frequency) were analyzed using a mixed model including diet, time and its interaction as fixed effects and Animal ID as a subject-specific effect considering random intercepts and random slopes for time. To consider the potential baseline imbalance between groups, the analysis was also adjusted considering the baseline variables: presence of blood (yes or no), fecal consistency, fecal odor and fecal frequency. The owner-reported dog apathy and vomiting were not significant in the model and were therefore excluded. Response variables based on binary outcomes (fecal odor and color) were analyzed using a generalized linear mixed model with a binomial distribution and a logit link with the same model terms. On both analyses, *post-hoc* comparisons were not corrected for multiplicity of contrasts. Odds ratios (OR) and relative risk (RR) were used to assess the association between exposure to Diet A or B and outcome of dysbiosis and normalization of various clinical signs. Of note, when they were not normalized in less than 9 days, the values were excluded to calculate the duration of the clinical signs.

In order to compare time to clinical signs’ normalization (fecal consistency <2), survival curves were obtained using the Kaplan–Meier estimator for censored data, these curves were compared by means of a Log-Rank test, and finally, the analysis was adjusted by the presence of blood using a PH-Cox regression model.

Statistical analysis was performed using SAS® v9.4 (SAS Institute, Cary, NC, United States). All statistical decisions were made using 0.05 as significance level.

## 3 Results

### 3.1 Animals and baseline evaluation

**Figure 2:**
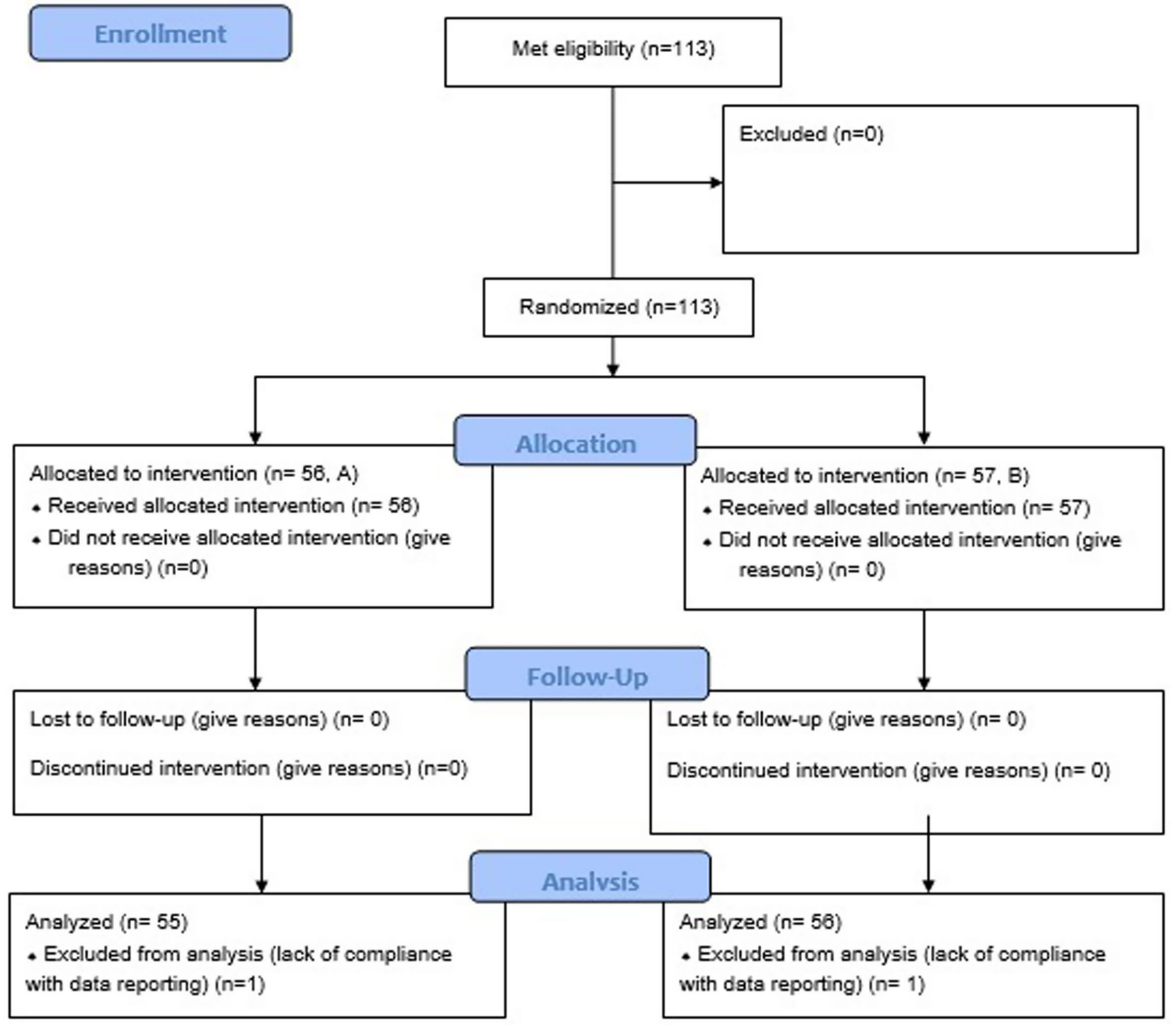
Clinical cases flow chart.

We recruited 113 dogs, with 56 dogs assigned to Diet A and 57 to Diet B. Two dogs (one in each group) were excluded from results analysis because of lack of compliance with data recording (Figure 2). No differences were observed between groups at baseline for mean age, body weight, BCS, breed, sex, number of days with clinical signs before presentation to the veterinarian, fecal odor or fecal color but 4 dogs (2 in each group) deviate from the target body weight range (1 < 4 kg and 1 > 45 kg in each group; Table 6). The majority of dogs can be considered as healthy in term of BCS (2.5 to 3.5/5, 66%) with a few proportions slightly overweight (22%) and a small proportion slightly underweight (11%). However, slight statistically significant differences were noticed for some fecal characteristics (consistency, frequency and quantity), with few clinical relevance (Table 6). The owner reported apathy, vomiting and blood-stained feces were notably more frequent in the control group than in the test group at baseline, which may be clinically relevant (Table 6).

**Table 6:** Group characteristics at baseline (T0) (Mean, Median, standard deviation SD, maximum, minimum and 95% confidence interval or absolute frequencies) and statistical differences observed between groups.

	Unit	Descriptive statistics	Diet A	Diet B	*p* value diet
Body weight	Kg	Mean	14.8	15.6	0.623
		Median	12.9	13.1	
		SD	9.8	9.6	
		Max	60.9	55.0	
		Min	3.2	3.7	
		Lower 95% CI	12.1	13.0	
		Upper 95% CI	17.4	18.1	
BCS	5-point score (1–5)	Mean	3	3	0.629
		Median	3	3	
		SD	0.6	0.7	
		Max	4	4	
		Min	2	1	
		Lower 95% CI	2.9	3.0	
		Upper 95% CI	3.2	3.3	
Age	Years	Mean	5.0	4.5	0.608
		Median	4	3	
		SD	3.3	3.2	
		Max	14	14	
		Min	1	1	
		Lower 95% CI	4.1	3.7	
		Upper 95% CI	5.9	5.3	
Sex	Male or female	Absolute %	Male 51% Female 49%	Male 54% Female 46%	0.713
Breed	description	Absolute %	data not shown	data not shown	0.230
Days before consultation	Days	Mean	0.4	0.4	0.865
		Median	0	0	
		SD	0.6	0.8	
		Max	2	4	
		Min	0	0	
		Lower 95% CI	0.2	0.2	
		Upper 95% CI	0.6	0.7	
Fecal consistency*	7-points score (−2–4)	Mean	3.5	3.3	0.017
		Median	3	3	
		SD	0.5	0.5	
		Max	4	4	
		Min	2	2	
		Lower 95% CI	3.3	3.1	
		Upper 95% CI	3.6	3.4	
Fecal frequency*	4-point score (0–3)	Mean	2.2	1.8	<0.001
		Median	2	2	
		SD	0.6	0.4	
		Max	3	3	
		Min	0	1	
		Lower 95% CI	2	1.7	
		Upper 95% CI	2.3	1.9	
Fecal quantity**	0–10	Mean	8.6	8.1	0.005
		Median	9	8	
		SD	0.7	1.1	
		Max	10	10	
		Min	7	3	
		Lower 95% CI	8.4	7.8	
		Upper 95% CI	8.8	8.4	
Fecal odor*	Abnormal	Absolute %	93% not normal	86% not normal	0.506
Fecal color*	Abnormal	Absolute %	98% not normal	98% not normal	1.000
Presence of blood	Yes	Absolute %	51%	21%	0.001
Presence of owner reported apathy	Yes	Absolute %	40%	7%	<0.001
Presence of vomiting	Yes	Absolute %	25%	1%	<0.001

^*^Fecal consistency, frequency, odor and color see Tables 2, 4 for explanation. **fecal quantity from 0 (very low) to 10 (very high).

### 3.2 Primary outcome: number of days with diarrhea

The number of days with low fecal consistency (score ≥ 2) was significantly lower with Diet B than with Diet A (Table 7). These numbers were calculated from dogs that achieved fecal consistence score < 2 within 8 days.

**Table 7:** Number of dogs included in each group and response to diets (absolute % or mean, median, standard deviation (SD), maximum, minimum and 95% confidence interval).

		Diet A	Diet B	*p*-value; diet effect
Number of dogs	Absolute number	55	56	-
Primary outcome				
Number of days with fecal consistency ≥2*	Mean	5.9	3.6	*p* < 0.001
	Median	6.0	3.0	
	SD	0.9	0.9	
	Max	8.0	6.0	
	Min	4.0	2.0	
	Lower 95% CI	5.6	3.4	
	Upper 95% CI	6.2	3.9	
Secondary outcome				
% dogs that improve in < 9 days (Fecal consistency <2)	absolute %	66%	98%	*p* < 0.0001 Odds Ratio = 28, *p* = 0.001; Relative Risk = 19, *p* = 0.003

*Calculated from dogs that clinically resolved (fecal consistency<2) in less than 9 days.

### 3.3 Secondary outcomes

#### 3.3.1 Survival curve (resolution of diarrhea)

**Figure 3:**
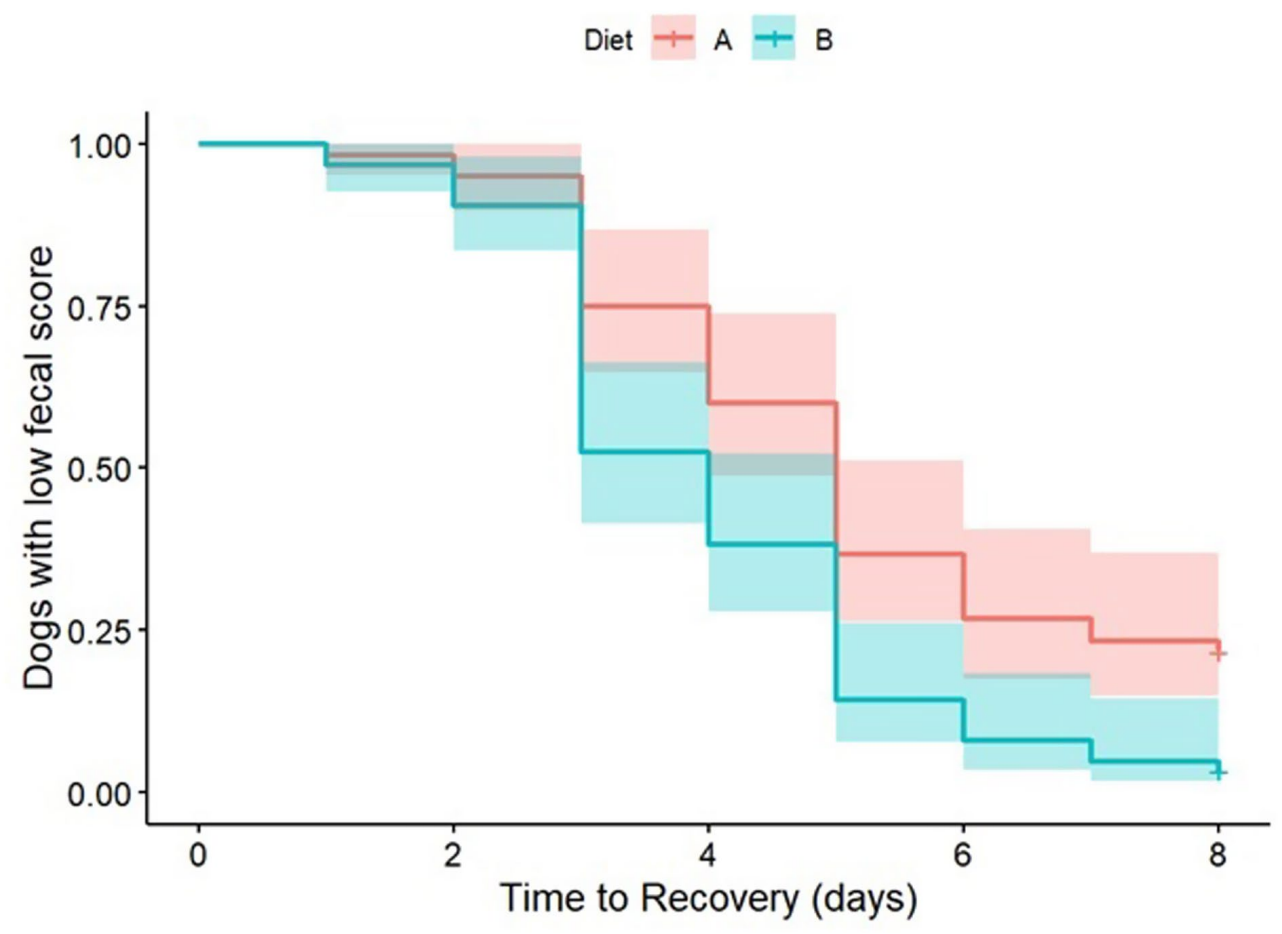
Kaplan–Meier survival curve estimated for censored data, with a Log-Rank test comparison, adjusted by the presence of blood using a PH-Cox regression model.

When analyzing the survival curve (with fecal consistency <2 considered as resolution of the diarrhea), a significant dietary effect (*p* = 0.032) was observed, with a hazard rate estimate of 0.64 between Diet B and Diet A, indicating that Diet B prescription resulted in a 1.6 times greater chance of resolving the clinical signs compared to Diet A (Figure 3). The presence of blood was not statistically significant in the model (*p* = 0.073).

#### 3.3.2 Percentage of dogs with recovery of normal fecal consistency score within 8 days

Most dogs (98%) fed Diet B needed fewer than 9 days to achieve a fecal consistency score of strictly less than 2 (≤1). In contrast, only 66% of dogs fed Diet A did so, leading to significantly higher OR and RR, indicating a quicker normalization of fecal consistency with Diet B (Table 7).

#### 3.3.3 Clinical secondary outcomes measured at each study days (Fecal characteristics)

**Figure 4:**
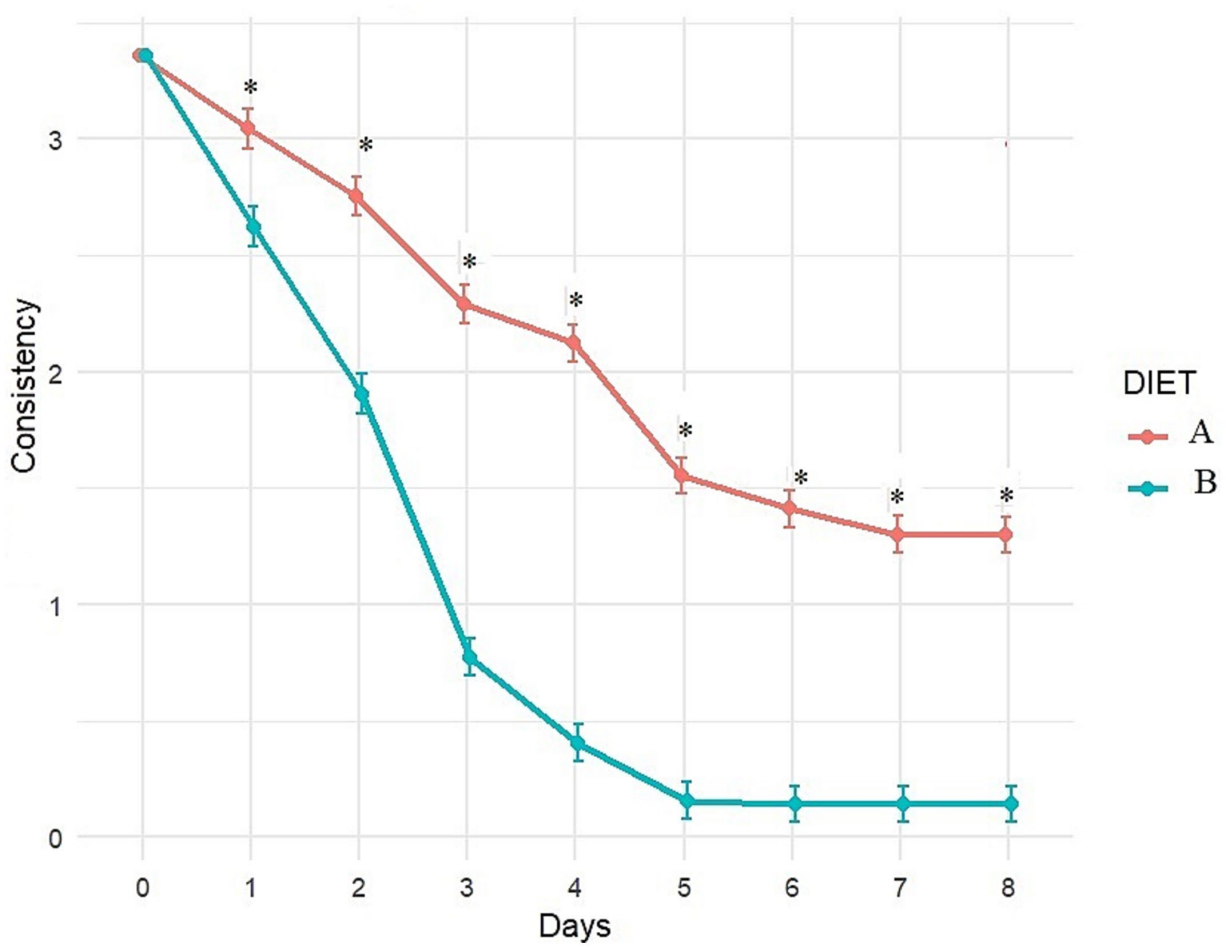
Evolution of fecal consistency (adjusted least square means estimates from mixed model) over time with Diet A (Control) and B (Test). A score of 0 and 1 indicates ideal consistency, 2 to 4 indicates mild to severe diarrhea. *Indicates significant difference between A and B.

**Figure 5:**
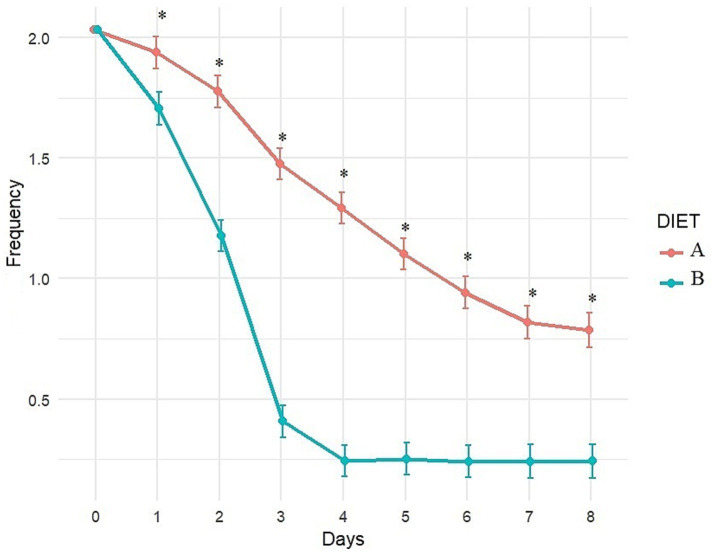
Evolution of fecal frequency (adjusted least square means estimates from mixed model) over time with Diet A (Control) and B (Test). A score of 0 indicates normal frequency, a score of 1 to 3 indicates mild to severe increase. *Indicates significant difference between A and B.

Fecal consistency and frequency were significantly better with Diet B compared to Diet A throughout the study (significant difference between diets from day 1 to day 8; Figures 4, 5). More precisely, a higher percentage of dogs fed diet B started to recover formed feces (consistency score = 2) after 1 day (44.6% vs. 3.6% with A, *p* < 0.0001, data not shown) and dogs fed diet B recovered firmer feces (average consistency score < 2) within 2 days. Dogs fed Diet A required 5 days on average for a similar outcome (consistency score <2; Figure 4). On average, normalization of frequency (score < 1) was attained with Diet B after 3 days, compared to 6 days with Diet A (Figure 5).

**Figure 6:**
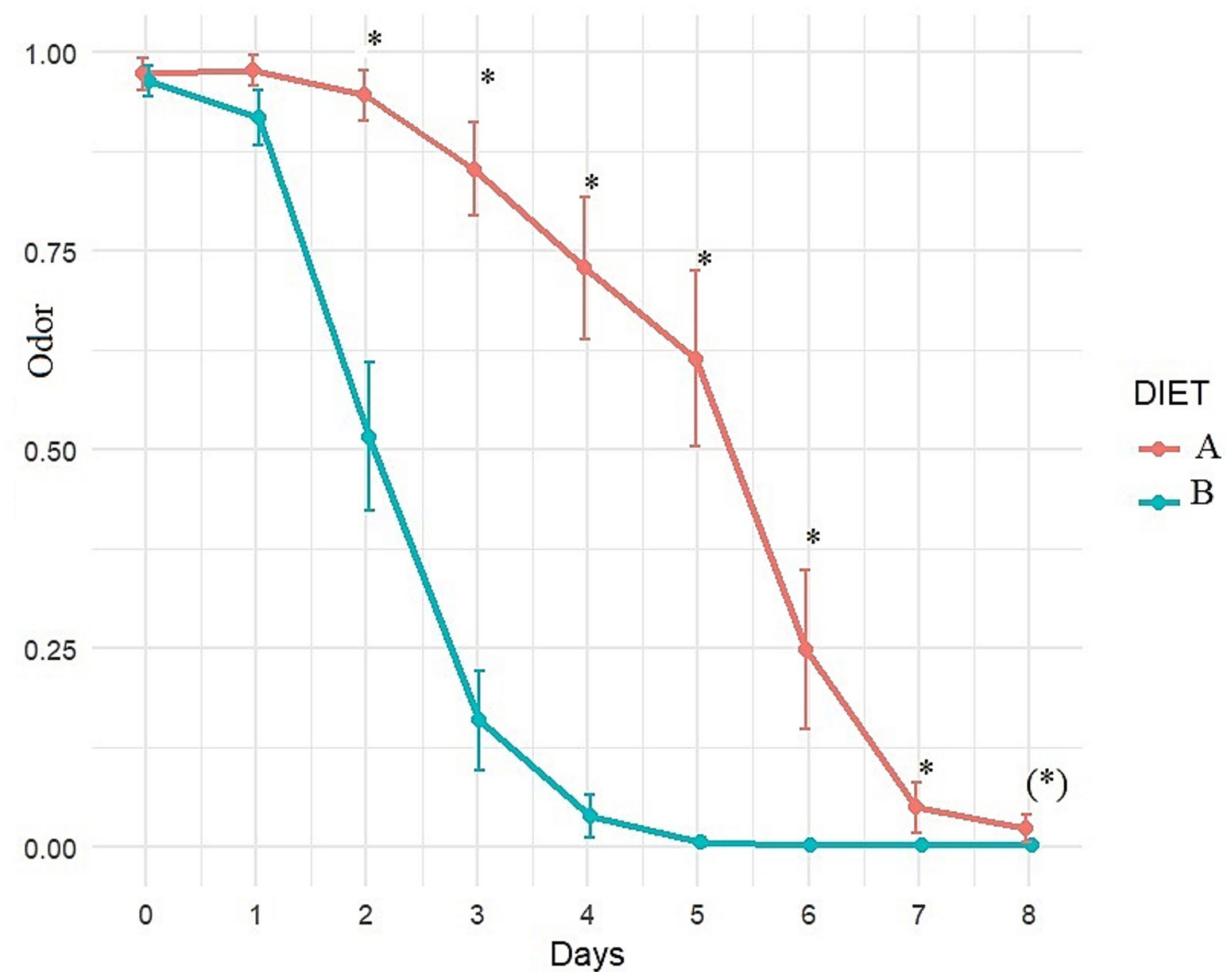
Evolution of fecal odor (adjusted least square means estimates from mixed model) over time with diet A (control) or B (Test). A score of 0 indicates normal odor, a score of 1 indicates abnormal odor. *Indicates significant difference between A and B and (*) indicates a trend.

Fecal odor showed significant improvement with Diet B compared to Diet A after 2 days of dietary treatment and continued to remain better throughout the follow-up period (Figure 6). Fecal odor improved significantly after 2 days with Diet B (*p* < 0.001) compared to T0, and after 3 days with Diet A (*p* = 0.034), compared to T0.

#### 3.3.4 Body weight and body condition score

At recheck, dogs with diet B maintained their body weight (15.6 ± 9.58 vs. 15.6 ± 9.64, *p* = 0.77) while dogs with diet A slightly lose some body weight (14.8 ± 9.83 vs. 14.6 ± 9.82, *p* < 0.0001). These changes of body weight were without significant impact on BCS.

#### 3.3.5 Satisfaction survey

Compliance with both diets was very high, with only two dogs dropped out of the study, for unrelated reasons. It was significantly easier to follow the recommendation with Diet B than with Diet A (7.7 ± 1.3 vs. 8.8 ± 0.7, respectively, *p* < 0.001). Globally, owners expressed significantly greater satisfaction with Diet B compared to Diet A, highlighted by a significantly higher satisfaction score (9.1 ± 0.4 vs. 6.3 ± 1.2, *p* < 0.001). In particular, diet B obtained a higher score for palatability that diet A (Table 8).

**Table 8:** Owners’ satisfaction survey about products and products characteristics [Mean, median, standard deviation (SD), maximum, minimum and 95% confidence interval (CI)].

		Product characteristics (from 0 to 10)							Global satisfaction	Product recommendation	Future choice
		Palatability	Easy to use	Satisfaction	Odor	Appearance	Texture	Color			
Scale		0–10	0–10	0–10	0–10	0–10	0–10	0–10	0–10	No 0/Yes1	No 0/Yes 1
Diet A	Mean	7.3	7.7	6.6	6.6	6.6	6.5	6.3	6.4	0.3	0.3
	Median	7.0	8.0	7.0	7.0	7.0	7.0	7.0	7.0	0.0	0.0
	SD	1.2	1.3	1.5	1.3	1.3	1.3	1.3	1.2	0.5	0.5
	Max	9.0	9.0	9.0	9.0	9.0	9.0	9.0	9.0	1.0	1.0
	Min	4.0	4.0	3.0	3.0	4.0	4.0	4.0	4.0	0.0	0.0
	Lower 95% CI	7.0	7.3	6.2	6.2	6.2	6.1	5.9	6.0	0.1	0.1
	Upper 95% CI	7.7	8.1	7.1	7.0	7.0	6.9	6.8	6.8	0.5	0.5
Diet B	Mean	8.7	8.8	8.4	8.1	7.8	7.8	7.8	9.0	1.0	1.0
	Median	9.0	9.0	9.0	9.0	8.0	8.0	8.0	9.0	1.0	1.0
	SD	0.7	0.7	1.3	1.6	1.5	1.5	1.4	0.4	0.0	0.0
	Max	10.0	10.0	10.0	10.0	9.0	9.0	9.0	10.0	1.0	1.0
	Min	6.0	6.0	2.0	2.0	2.0	2.0	3.0	8.0	1.0	1.0
	Lower 95% CI	8.5	8.6	8.1	7.6	7.4	7.4	7.4	8.9	1.0	1.0
	Upper 95% CI	9.9	9.0	8.8	8.5	8.2	8.2	8.2	9.2	1.0	1.0
*p-*value diet		< 0.001	< 0.001	< 0.001	< 0.001	< 0.001	< 0.001	< 0.001	< 0.001	< 0.001	< 0.001

The veterinarians were also very satisfied with the use of Diet B, giving it a score of 9.1 ± 0.5 for effectiveness (vs. 6.1 ± 0.5 for Diet A, *p* < 0.001), and all of them stated they would recommend Diet B in the future.

#### 3.3.6 Fecal dysbiosis index

At T0, fecal analysis revealed that 39% of dogs fed with Diet A and 47% of dogs fed with Diet B had some degree of dysbiosis (mild or severe), with no statistical difference between groups (Table 9).

**Table 9:** Number of dogs with and without dysbiosis (absolute %), DI and targeted bacterias analysis (log DNA/g; mean + − SD) at T0 and at T1.

	Unit	Descriptive statistics	Diet A		Diet B		*p*-value diet effect		*p*-value time effect		Odds ratio and relative risk diet effect		Odds ratio and relative risk time effect	
			T0	T1	T0	T1	T0	T1	Diet A	Diet B	T0	T1	Diet A	Diet B
Dogs without dysbiosis (DI < 0)	% dogs	Absolute %	61	39	53	71	0.255	<0.0001	0.002	0.0009	RR 1.2, *p* = 0.389 and OR 1.4 *p* = 0.390	RR = 0.49, *p* = 0.002 and OR = 0.27, *p* = 0.001	RR 1.6 *p* = 0.03 and OR 2.4 *p* = 0.02	RR = 0.63, *p* = 0.06 and OR = 0.47, *p* = 0.056
Dogs with dysbiosis (D I > 0)	% dogs	Absolute %	39	61	47	29								
*Mild dysbiosis (DI = 0–2)*	*% dogs*	Absolute %	*20*	*27*	*19*	*10*	-	-	-	-	-	-	-	-
*Severe dysbiosis (DI>2)*	*% dogs*	Absolute %	*20*	*34*	*28*	*19*	-	-	-	-	-	-	-	-
Dysbiosis (DI)	-	Mean	−0.42	0.65	−0.49	−1.05	0.968	0.002	0.09	0.304	-	-	-	-
		Median	−0.65	0.75	−0.50	−1.40					-	-	-	-
		SD	3.35	3.12	3.40	3.03					-	-	-	-
		Max	7.40	7.30	6.50	5.80					-	-	-	-
		Min	−6.69	−6.20	−6.40	−5.80					-	-	-	-
		Lower 95% CI	−1.31	−0.18	−1.40	−1.85					-	-	-	-
		Upper 95% CI	0.48	1.49	0.42	0.24					-	-	-	-
Specific bacteria (reference range)														
*Blautia* spp. (9.5–11.0)	log DNA/g	Mean	9.98	9.86	9.95	9.93	0.752	0.660	0.495	0.797	-	-	-	-
		Median	10.10	10.00	10.00	10.10					-	-	-	-
		SD	0.78	0.79	0.79	0.55					-	-	-	-
		Max	10.90	10.90	11.00	10.80					-	-	-	-
		Min	7.00	7.30	8.10	8.20					-	-	-	-
		Lower 95% CI	9.77	9.65	9.76	9.79					-	-	-	-
		Upper 95% CI	10.19	10.07	10.13	10.07					-	-	-	-
*Peptoacetobacter* (formerly *Clostridium*) *hiranonis* (5.1–7.1)	log DNA/g	Mean	5.39	4.29	5.10	5.18	0.316	0.956	0.058	0.880	-	-	-	-
		Median	6.30	6.05	6.10	6.10					-	-	-	-
		SD	2.17	2.79	2.43	2.32					-	-	-	-
		Max	7.20	7.10	7.20	7.40					-	-	-	-
		Min	0.10	0.10	0.10	0.10					-	-	-	-
		Lower 95% CI	4.80	3.54	4.46	4.56					-	-	-	-
		Upper 95% CI	5.97	5.04	5.74	5.79					-	-	-	-
*E. coli* (0.9–8.0)	log DNA/g	Mean	5.93	6.43	5.78	5.93	0.789	0.254	0.223	0.585	-	-	-	-
		Median	6.35	7.10	5.90	6.00					-	-	-	-
		SD	1.92	1.93	1.96	2.08					-	-	-	-
		Max	9.10	9.20	8.90	8.60					-	-	-	-
		Min	1.60	1.70	1.60	1.70					-	-	-	-
		Lower 95% CI	5.42	5.91	5.25	5.38					-	-	-	-
		Upper 95% CI	6.45	6.95	6.30	6.48					-	-	-	-
*Faecalibacterium* spp. (3.4–8.0)	log DNA/g	Mean	5.00	4.76	5.24	4.63	0.187	0.556	0.107	0.002	-	-	-	-
		Median	5.20	4.70	5.30	4.60					-	-	-	-
		SD	0.99	1.07	0.93	1.33					-	-	-	-
		Max	6.50	6.60	6.90	6.90					-	-	-	-
		Min	1.90	2.20	3.30	1.10					-	-	-	-
		Lower 95% CI	4.74	4.47	4.99	4.27					-	-	-	-
		Upper 95% CI	5.27	5.05	5.49	4.98					-	-	-	-
*Fusobacterium* spp. (7.0–10.3)	log DNA/g	Mean	8.54	8.25	8.49	8.36	0.906	0.768	0.046	0.310	-	-	-	-
		Median	8.50	8.30	8.50	8.30					-	-	-	-
		SD	0.99	0.93	0.79	0.86					-	-	-	-
		Max	10.10	10.50	9.90	9.80					-	-	-	-
		Min	6.80	6.20	6.90	6.50					-	-	-	-
		Lower 95% CI	8.27	8.00	8.28	8.14					-	-	-	-
		Upper 95% CI	8.80	8.50	8.70	8.59					-	-	-	-
*Streptococcus* spp. (1.9–8.0)	log DNA/g	Mean	5.71	5.54	5.54	4.95	0.836	0.134	0.593	0.019	-	-	-	-
		Median	5.90	5.00	5.40	4.50					-	-	-	-
		SD	1.84	1.66	1.80	1.70					-	-	-	-
		Max	8.50	8.70	8.70	8.80					-	-	-	-
		Min	2.20	2.60	2.20	2.10					-	-	-	-
		Lower 95% CI	5.22	5.09	5.06	4.49					-	-	-	-
		Upper 95% CI	6.20	5.98	6.02	5.40					-	-	-	-
*Turicibacter* spp. (4.6–8.1)	log DNA/g	Mean	6.23	6.43	6.15	6.39	0.838	0.720	0.197	0.113	-	-	-	-
		Median	6.20	6.30	5.90	6.40					-	-	-	-
		SD	1.13	1.15	1.15	0.97					-	-	-	-
		Max	8.20	8.90	8.50	8.10					-	-	-	-
		Min	3.00	4.40	3.80	3.90					-	-	-	-
		Lower 95% CI	5.92	6.16	5.85	6.13					-	-	-	-
		Upper 95% CI	6.53	6.69	6.46	6.64					-	-	-	-

At recheck (T1), the number of dogs with mild or severe fecal dysbiosis increased with Diet A (61% at T1 vs. 39% at T0) and decreased with Diet B (29% at T1 vs. 47% at T0) compared to T0 (Table 9). These changes were significant in both groups. That means that at T1, dogs fed Diet B demonstrated a lower risk and lower odds of having fecal dysbiosis compared to dogs fed Diet A. Specifically, the Relative Risk (RR) was 0.49 (*p* = 0.0001), indicating a 51% reduction in risk. The Odds Ratio (OR) was 0.27 (*p* = 0.001), signifying a 73% reduction in odds (Table 9). Regarding the quantification of the bacterias used to calculate the DI, at T0, the numbers of DNA copies of *C. hiranonis* (21% of the cases, 23% with A and 21% with B), *Blautia* (19% of the cases) and *E. coli* (14% of the cases) were the ones found more frequently out of the reference range (data not shown).

Few relevant differences were observed on the different bacterias with time or with diet. A trend to decrease *C. hiranonis* at T1 was observed with diet A, while a numerical increase was observed with diet B. On average, all the bacterias were in reference range except *C. hiranonis* at T1 with diet A. At T1, the % of dogs with low *C. hiranonis* was 36% with diet A (increased compared to T0) and 21% with diet B (same as at T0; data not shown).

The presence of fecal dysbiosis at T0 increased the OR and the RR for not achieving the fecal consistency normalization (consistency score < 2) with Diet A (OR 5.8, *p* = 0.006; RR 3.4, *p* = 0.009) but had no effect with Diet B (data not shown). Some of the dogs that still exhibited gastrointestinal clinical signs and/or dysbiosis at rechecking were switched to Diet B (*n* = 39). Dysbiosis resolved in half of these dogs (*n* = 19) after a longer period of feeding with Diet B. All the dogs that continued to have low fecal scores showed clinical improvement with Diet B (Data not shown).

## 4 Discussion

The primary objective of this double-blind, controlled study was to evaluate whether a highly digestible diet containing one single strain of probiotic, FOS, SDPP, yeast and sepiolite would help resolve clinical signs associated with mild acute diarrhea in dogs more quickly compared to a highly digestible diet without supplementation.

To achieve our goals, we recruited 113 dogs suffering from acute diarrhea and allocated them to one diet or the other using a randomization list with a 1:1 ratio. At baseline, no significant difference was obtained for the dogs’ body weight, BCS, age, sex, breed, or the number of days with clinical signs before visiting the veterinarian. The majority of dogs can be considered as healthy in term of BCS (2.5 to 3.5/5, 66%) with a few proportions slightly overweight (22%) and a small proportion slightly underweight (11%). This overweight proportion is representative of the general canine population in Spain, although some bias can be present in the study of Muňoz-Prieto et al. as BCS were estimated by owners (48). We found no other data on the percentage of underweight dogs in Spain but it slightly exceeds % reported in France (4.7%) (49). In terms of clinical signs, fecal odor and color did not differ between the two groups. However, fecal consistency, frequency, and quantity showed statistically significant differences (though not deemed clinically relevant), with less consistent feces observed in dogs fed Diet A vs. Diet B. Furthermore, the presence of blood, vomiting events, and apathy were more frequent in dogs fed Diet A than Diet B. A study on acute hemorrhagic diarrhea in dogs reported that vomiting preceded the onset of bloody diarrhea in 80% of dogs (50). This observation agrees with our finding that bloodier feces coincided with vomiting in the Diet A group. Based on these elements, it is difficult to definitively conclude whether the acute diarrhea was more severe in dogs fed Diet A vs. Diet B. Indeed, another study indicated that most dogs hospitalized with suspected acute hemorrhagic diarrhea do not require antimicrobial treatment, even when showing signs of systemic disease on initial presentation and typically recover quickly (51). These minor initial differences might only suggest different etiologies among the dogs, reflecting the broad spectrum of acute diarrhea events. In both groups, despite the slight baseline differences, we were primarily dealing with dogs presenting mild acute hemorrhagic and non-hemorrhagic diarrhea. We therefore assumed the clinical conditions were appropriate for testing the efficacy of the diets.

Our results demonstrated that the administration of the tested diet accelerated the normalization of stool consistency, frequency and odor, compared to control, with a reduction in the number of days with clinical signs (−2.3 days with low fecal consistency, calculated from dogs that clinically improved in less than 9 days, 66% on Diet A vs. 98% on Diet B) with 1.6 times better resolution rate. The magnitude of the effect is considered moderate according to the ENOVAT guidelines and thus might be considered as clinically relevant (9). While few robust studies have been designed to evaluate the impact of a diet on acute diarrhea—with most reports focusing on chronic diarrhea—our results are comparable to or even surpass those of other published studies. These studies, which examined probiotic and/or nutraceutical supplementation in dogs with acute diarrhea, reported a decrease of 0.63 to 2.7 days in the number of days with low fecal scores (52–56). Nixon et al. (55) reported an estimate of 1.6 from survival curves when they tested a paste containing *E. faecium* 4b1707, a prebiotic, combined kaolin and montmorillonite clay, psyllium, pectin, and beta-glucan, in line with our results too. In their meta-analysis, Scahill et al. (57) reported that pro-, pre-, and synbiotics had a smaller effect, between 0.62 and 1.2 days reduction of diarrhea duration. Different authors also studied the use of fiber. Lappin et al. (41) reported that a high fiber diet (as fed: 4.54% soluble and 15.16% insoluble fiber vs. 0.6 and 5.33% in the control diet) resulted in improving the fecal score of shelter dogs subjected to acute large bowel diarrhea followed for 9 days. Holz et al. (13) reported a modest effect in dogs with acute diarrhea fed with a gastrointestinal diet and supplemented with a powdered cellulose at a dosage of 0.5 g/kg of body weight/d, with a trend for an improvement of the fecal score on day 1. Interestingly, Rudinsky et al. (58) demonstrated that the management of the acute diarrhea with a dedicated diet was more effective than a treatment with metronidazole, emphasizing the fact that nutritional management may be the most efficient strategy, and is more sustainable with regards to antimicrobial resistance issue. This diet contained 28.3 g total dietary fiber/Mcal, reached thanks to psyllium husks. Lee et al. (59) did use a blend of fiber, pre-, yeast-based postbiotics and SDPP and observed the improvement of fecal consistency in healthy dogs, suggesting that this combination can effectively act on feces quality. Based on those results, it can be concluded that adding clay, prebiotic (FOS), fiber (cellulose), and probiotic represent a good strategy to manage acute diarrhea. In addition to that, our combo was also designed with SDPP and yeast. Based on our current results and on the literature data, we assume that our specific diet was efficient to faster the normalization of the fecal consistency in dogs suffering from acute diarrhea. The amplitude of the answer, stronger than in the meta-analysis of Scahill et al. (57) and aligned with the study of Nixon et al. (55) suggests that a mixture of various nutraceuticals combined with highly digestible ingredients is more efficient than the use of a pre-, pro- or synbiotics alone, confirming that the use of a specific complete and balanced diet is an adapted tool to manage this situation.

Dogs fed the test diet maintained their body weight, whereas those receiving Diet A experienced a slight decrease. Given the short duration of the study, this may be considered relevant. This observed difference in body weight may be attributed to a more rapid recovery associated with Diet B compared to Diet A. Alternatively, it could reflect differences in disease etiology and/or severity. Feeding guidelines were established using identical equations to calculate the metabolizable energy of both diets and the dogs’ energy requirements in both groups. The quantity of food offered remained constant throughout the trial. Owners reported that Diet B was more palatable than Diet A, which may have contributed—at least partially—to the outcomes observed as well. It is also possible that energy needs have been underestimated in some animals. The recommended quantity of food was established with the veterinarian, based on the activity provided by the owner, but owners were responsible for weighting the food themselves- which can result in mistakes-, and leftover food was not recorded. This lack of data limits the ability to fully interpret the results and should be considered a limitation of the study.

The results of our study revealed that Diet B might exert a direct effect on gut microbiota. Indeed, we found that the presence of fecal dysbiosis at T0 increased the risk of not achieving fecal normalization with Diet A but not with Diet B. Recent evidence suggests that the fecal microbiota composition is altered in dogs with acute diarrhea (17, 60). Recent studies showed, using 16S rRNA sequencing, that the microbiome of dogs was significantly modulated when suffering from acute diarrhea with notably an overrepresentation of *Clostridium perfringens* while decreasing the relative abundance of SCFAs producing bacteria like *Faecalibacterium, Blautia* or *Turicibacter* (17, 60). To evaluate the status of the microbiome, we analyzed the fecal DI, a quantitative PCR-based test used to detect intestinal dysbiosis (16). This DI has been initially developed for dogs suffering from chronic enteropathies; however, it has also been used in cases of acute diarrhea (13, 61). In our study, at T0, 39% of dogs fed with Diet A and 47% of dogs fed with Diet B exhibited slight to severe degrees of dysbiosis, primarily associated with low levels of *C. hiranonis* (21% of cases), low levels of *Blautia* (19% of cases), and high levels of *E. coli* (14% of cases). Holz et al. (13) found a moderate degree of dysbiosis in dogs with acute diarrhea, primarily due to an increase in *C. perfringens* and *E coli*. In our study, Diet B contributed to a restoration of the richness and/or evenness. Indeed, the DI is supposed to negatively correlate with species richness (61), meaning that a higher one indicates lower microbial diversity. Thus, following this assumption, a proportion of the dogs that were recruited in our study harbored lower diversity (either richness or evenness). This agrees with the results reported by Chaitman et al. (61), who observed a decrease in alpha-diversity, while an increase in DI in dogs suffering from acute diarrhea when compared to healthy animals’ microbiota. It is worth noting that one of the most affected bacteria was *C. hiranonis*, a major bile acid-converting *bacterium* which allows the conversion of primary biliary acids into secondary biliary acids. In agreement with our results on gut microbiota composition, acute diarrhea has been associated with changes in the microbiome activity (62), among which are a change in biliary acid metabolism (61). Of note, those molecules have antimicrobial effects that may decrease the growth of potential enteropathogens such as *C. perfringens,* which is a common cause of acute diarrhea (13). However, a quantification of *C. perfringens* and *C. difficile* would have been interesting to further support this potential mode of action.

Evidence suggests that probiotics and prebiotics can alter the microbiome composition of dogs (21, 30–32, 35, 63–70). More specifically, the dietary supplementation of dogs with *Bacillus velezensis* has been shown to increase gut bacterial diversity and the abundance of beneficial bacterial groups for gut health, such as *Bacteroides, Faecalibacterium,* and *Allobaculum*, compared to control (21, 71, 72). Prebiotics, such as FOS, also play a crucial role in maintaining a balanced intestinal microbiota. For instance, adding FOS to the canine diet increased the number of probiotic bacteria, including *Bifidobacterium* and *Lactobacillaceae family,* while reducing the number of *C. perfringens* (70). In addition, SDPP has been shown to change gut microbiota composition by promoting the presence of Firmicutes, and specifically the lactobacilli population (28). It also enhanced the growth of species involved in regulatory T-lymphocyte homeostasis and restoration of the mucosal barrier, as well as species negatively correlated with expression of pro-inflammatory cytokines in healthy mice, suggesting a microbiota-mediated effect of SDPP (28).

Acute diarrhea is also usually associated with a stronger proteolytic activity, a decrease in starch and glucose metabolism and an increase in gut permeability (62). This may lead to the increased formation of putrefactive compounds, softer feces and bad smell. It is highly probable that Diet B allowed a quicker restoration of the microbiota activity, as suggested by the significantly faster decrease in bad odors. In agreement with that, several authors reported that the dietary supplementation of healthy dogs with *B. velezensis* reduced fecal ammonia content while decreasing fecal odors (21, 71, 72). In addition, Lee et al., using a blend of fiber, prebiotics, yeast-based postbiotics and SDPP obtained a drastic decrease in fecal branched chain fatty acids, isovalerate, isobutyrate, phenol, and ammonia concentrations in healthy dogs (59). Similarly, the use of FOS has been shown to increase fibrolytic activity while decreasing proteolytic activity, and, in turn, both fecal odors and putrefactive compounds, among which, ammonia, indoles and phenols (32). Soluble fiber might increase the production of lactate and acetate thanks to homo- and hetero-fermentative *Lactobacillaceae* and *Bifidobacteriaceae*, and further stimulated the growth of propionate and butyrate producers’ bacteria through cross feeding (73). Butyrate is largely used as an energy source for colonocytes and may improve gut permeability, thus contributing to a quicker restauration of the gut homeostasis. However, we did not measure the fecal concentrations of SCFAs and putrefactive compounds, and this would further require confirming this assumption.

Besides effect on the gut microbiome, Diet B might result in other effects to faster normalize the fecal consistency. First, a change in osmolarity of the gut thanks to the addition of sepiolite could occur. Sepiolite is a fibrous clay mineral that differs from laminar clays by having tunnels in its structure. These tunnels can hold water as well as other small molecules, giving to this clay good absorption capacities (74). Although sepiolite has been studied in less extent than other types of clay like smectite or montmorillonite, it has been suggested that sepiolite could mitigate diarrhea produced by toxins or specific amines, thanks to its ammonia and amines absorption, reducing the bad smell as well (39). As an example, Elitok and Baser demonstrated that sepiolite might be efficient to treat calf’s diarrhea (75). Second, we cannot exclude an action of Diet B could through a direct effect on the host’s cells. It has been demonstrated that the oral administration of IgG from SDPP in dogs maintains its activity throughout the digestive system, suggesting it transfers passive immunity at the intestinal level in dogs, contributing to their natural defenses (24). Moreover, it has also been shown that IgG from SDPP improves gut immunity *in vitro*, specifically by decreasing the enteropathogen adherence with the cells, indicating a potential protective gut effect in dogs (25). Lee et al. observed higher content of fecal secretory IgA, and a greater ratio between T helper cells and cytotoxic T cells with the blend of fiber, biotics and SDPP (59). Peace et al. demonstrated that the use of SDPP (2.5 and 5% inclusion) for weaned piglets was efficient to trigger the gut permeability and decrease IFNγ pro-inflammatory cytokines (76). Probiotics, which harbor many surface layer proteins in their bacterial wall, are also able to interact directly with the host’s cells through a crosstalk, leading to a change in the gut homeostasis (77). The direct effects of FOS on intestinal immunity have been recently reviewed, showing that those fiber may activate receptors present in the immune cells and resulting in anti-inflammatory effects (78).

It is also difficult to estimate to which extent the difference in macronutrients and ingredients between diets contributed to the clinical outcomes. The impact of decreasing the fat content in the test diet on clinical outcomes remains unclear. Fat delays gastric emptying. Assimilation of dietary fat is a relatively complex process, and mal-absorbed fatty acids are hydroxylated by intestinal and colonic bacteria. Hydroxy–fatty acids stimulate colonic water secretion and exacerbate diarrhea and fluid loss. Fat malassimilation can also be associated with malabsorption of bile acids, resulting in deconjugation of unabsorbed bile acids and increased mucosal permeability and secretion (79). However, in association with highly digestible diet, fat is generally well tolerated across most canine digestive diseases (80, 81). The inclusion of coconut oil did not increase fat digestibility in diet B compared to diet A as it could have been expected, indirectly suggesting the high quality of the control diet’s fat source (82). While positive effects from fish oil (anti-inflammatory) would be expected at medium term, it is not clear if they can contribute significantly to the short-term outcomes measured in the study (83). A significant variable lies in the fiber sources. The control diet contained 2.7% sugar beet pulp, which benefits gut health (84), while the test diet used a different blend of cellulose and FOS as commented above. The differential effects of these specific fiber types are complex to disentangle. Dried whole yeast (1%) was also added in the tested diet, with a potential effect on gut microbiota, gut barrier and immune system through MOS and Beta-glucans (85, 86). A recent study suggested that concentrated brewer’s yeast may have the potential to reduce gut permeability without impacting inflammatory status and markers of health in adult dogs (85). The two diets differed slightly in their protein content and were formulated using distinct protein sources. In dogs with acute diarrhea, increased protein digestibility may result in less undigested protein reaching the colon, thereby mechanically reducing microbial fermentation. Although Diet B showed a numerically higher protein digestibility, the difference was not statistically significant. Consequently, it remains unclear whether the observed outcomes were influenced by differences in protein quantity, sources, or both.

Our study pinpointed out the difficulty to “categorize” diarrhea. Due to its self-limiting nature, clinical recovery of acute diarrhea typically occurs within 1 week, but we have seen that not all the cases enrolled in our study recovered a normal fecal consistency after 8 days. Indeed, some of the dogs fed Diet A and exhibiting clinical signs and/or dysbiosis at recheck were transitioned to Diet B, which resulted in dysbiosis resolution in half of these cases with longer feeding periods; however, this was not achieved in all cases. The timeframe allowed for clinical improvement (less than 9 days, which translates to 8 days following inclusion) has been arbitrarily determined and corresponds to clearly acute diarrhea (lasting 3 to 7 days). However, if diarrhea is generally considered chronic after 21 days (87), there is a “grey zone” between 8 and 20 days, defined as “prolonged” in human medicine (88, 89). The lower percentage of dogs that resolved clinical signs (fecal score < 2) in less than 9 days with Diet A compared to Diet B was surprising. It is possible that, despite the blinded randomization, more severe or chronic cases were assigned to Diet A than to Diet B. Indeed, as discussed before, some clinical signs were more pronounced at baseline in group A than in group B. It is also possible that a small percentage of dogs with chronic or “prolonged” (8–20 days) diarrhea respond better to Diet B than to Diet A, as the formulation and functional ingredients included in Diet B are designed for dogs with chronic enteropathies as well (e.g., probiotics and prebiotics).

Our double-blinded, randomized, controlled study has been designed to demonstrate the efficacy of a full diet – and not only nutraceuticals - to accelerate the normalization of the fecal consistency of dogs suffering from uncomplicated diarrhea. A key strength of the study was that veterinarians assessed the fecal consistency at baseline and at the end of the follow-up with a recognized scale. Overall, our results showed that a highly digestible diet made with highly digestible ingredients and containing a probiotic, namely *B. velenzensis,* combined with FOS, SDPP, yeast and sepiolite, can faster the normalization of the fecal consistency in dogs suffering from acute diarrhea, and is thus a useful tool for the vet practitioner to manage this common disease, avoid the use of antibiotics while resulting in visible signs for the owner and a quicker recovery for the dog. Several complementary modes of action might be implied to explain this result, with, notably, a modulation of the gut microbiota composition and activity, and a direct effect on the gut barrier. However, the current results do not clarify the extent to which each active ingredient contributed to the positive effect observed for the tested diet. Consequently, further work is necessary to elucidate the mechanism of action of the tested diet.

The present results should be interpreted cautiously as they present several limitations. First, we wanted to compare two highly digestible complete and balanced diets with similar macronutrients digestibility. It is unclear whether the effects observed in the control group are due to specific nutrients/ingredients or the overall composition of the control diet, and whether the positive effects seen in the test group are solely attributable to the synbiotics mix, or if other dietary factors are contributing as commented before. In addition, it was assumed that the control diet would not affect the outcome of acute diarrhea. However, differences in the macronutrient profiles (i.e., fat and fiber) and ingredients of the tested diet and control could have influenced the duration of clinical symptoms and/or the dysbiosis index as commented before (55). Second, the use of a complex dietary formulation also limits the extent to which our findings can be extrapolated to other similar dietary treatments. Small differences in either the ingredients, nutrients, or active components could affect the efficacy of the dietary treatment. Third, dogs enrolled in the study can be considered to have mild clinical signs (absence of fever, no detectable dehydration, no systemic signs); therefore, the interpretation of the results must be limited to these cases and should not be extrapolated to more severe or chronic illnesses. Similarly, although we enrolled a quite high number of dogs, they were from the same geographical region. Forth, other fecal characteristics than first and final fecal consistency were evaluated by owners and owners were responsible of the given quantity of food, which may present a limitation. Finally, while the current data suggest potential benefits of the diet in managing the signs of acute diarrhea in dogs, the absence of blood and fecal analyses represents a notable gap in the evidence base. Furthermore, research into the mode of action of the included nutraceuticals, particularly through studies using canine-specific cell lines, would provide valuable mechanistic insights. Such *in vitro* models could help elucidate how bioactive compounds interact with intestinal or immune cells as well as with specific canine pathogens, thereby supporting the interpretation of clinical outcomes.

## 5 Conclusion

In conclusion, this randomized, double-blind, controlled trial demonstrated that a highly digestible diet supplemented with probiotic, FOS, SDPP, yeast and sepiolite (Diet B) significantly outperformed a highly digestible control diet (Diet A) in the management of acute uncomplicated mild canine diarrhea. Diet B resulted in faster clinical resolution (−2.3 days on average), improved fecal consistency, frequency, and odor, and in a reduced incidence of dysbiosis compared to Diet A. Notably, Diet B led to a significantly higher rate of clinical normalization within 8 days (1.6 times more). Furthermore, owners and veterinarians reported greater satisfaction with Diet B, highlighting its ease of use and efficacy. These findings suggest that Diet B represents a superior dietary approach for the rapid and effective management of mild acute canine diarrhea.

## Data Availability

The raw data supporting the conclusions of this article will be made available by the authors, without undue reservation.
